# Editorial: Surgical Management Options in Proximal Hypospadias

**DOI:** 10.3389/fped.2019.00128

**Published:** 2019-04-11

**Authors:** Ricardo González, Miguel Alfedo Castellan

**Affiliations:** ^1^Pediatric Surgery and Urology, Kinder- und Jugendkrankenhaus AUF DER BULT, Hanover, Germany; ^2^Pediatric Urology, Miami Children's Hospital, Miami, FL, United States

**Keywords:** hypospadias, complications, urethrocutaneous fistula, onlay repair, metal based flap

This research topic intended to highlight different philosophies and techniques for the correction of proximal hypospadias, a topic that continues to generate great controversy among pediatric urologists. Unfortunately, there were only four papers accepted to be included in this collection.

One paper dealt with the results of one-stage repair of mid- and proximal hypospadias, a second paper dealt with a technique to facilitate second-stage repairs when the urethral plate created in the first stage with a free graft was difficult to tabularize, a third paper reviewed the current status of tissue engineering for urethral reconstruction, and the fourth one dealt with the results of a modified Mathieu technique for repair of the megameatus/intact prepuce variant of hypospadias.

Authors from Germany (Hannover and Berlin) reported the results (measured by reoperation rate) in 49 children operated with a technique first described by the senior author in 1996 and followed for 2 years after the operation (González et al.). The technique consists of the preservation of the urethral plate whenever possible (48 of the 49 patients) and then creating an onlay urethroplasty using the outer preputial layer of a total preputial flap transposed ventrally through a buttonhole in its pedicle to avoid rotation. The penile curvature was corrected with chordectomy alone in 10 patients; 38 patients required a dorsal plication of the tunica albuginea and 1 patient required an additional ventral dermal graft. Success, defined as a straight penis, a glanular meatus, and the absence of voiding symptoms, was achieved in 77.5% of the patients with a single procedure. Complications included urethrocutaneous fistulas in 16% of the patients, glans dehiscence in 6% of the patients, and recurrent chordee in one patient. Of the 13 patients that had uroflow examinations, 11 had bell-shaped curves. This report would suggest that one-stage repairs can achieve good results with fewer operations than the more commonly used staged techniques. Shortcomings of this report include relative short follow-up and lack of objective cosmetic evaluation.

A group from Vienna reported on 35 consecutive patients from a cohort of 250 hypospadias cases who completed both steps of a staged repair (Tonnhofer et al.). The original idea in this report, however, is based on the fact that in two-thirds of the patients, a midline incision of the graft was considered necessary to achieve tubularization during the second stage. With a mean follow-up of 18 months, the complication rate was 22%, similar to that reported in the previously commented paper for a single-stage repair. The complication rate in this series was greater with the use of buccal mucosa than with the use of a free preputial graft. Complication rate in cases in which the plate was not incised (presumably because it was wider or of better “quality”) was just 8.3%. Despite these results, the authors conclude that a two-stage repair is the procedure of choice in the correction of severe hypospadias. Other shortcomings of this report include short follow-up, lack of objective criteria to decide to incise the plate, and lack of objective evaluation of the results of the urethroplasty, such as uroflow studies or calibration. Therefore, this paper adds a trick to facilitate the second stage, but the authors fail to demonstrate the real value of the maneuver. Perhaps it would be more productive to emphasize the need to have a very generous graft in the first stage and to wait until the graft is soft and pliable to perform the second stage.

A surgeon from Boston describes his experience with the use of a metal-based flap to repair the megameatus intact prepuce variant of hypospadias (MIP) (Cendron). Twenty-five patients operated in a 10-year period were divided into two groups. The first 10 cases underwent simple tubularization of the plate. One fistula developed. The next 15 cases underwent repair using a metal-based flap. There were no fistulas, but one patient experienced dehiscence of the glans. The author favors the metal-based flap technique, because in this series, it eliminated injury of the urethral plate during the dissection. Of course, the numbers are too small to draw definitive conclusions, but given the rarity of this condition, the author's opinion is worth considering.

A paper by authors from Qatar and Denmark analyzed the current knowledge of tissue-engineered material for urethral replacement (Abbas et al.). The clinical experience cited is limited, but tissue engineering will certainly become a reality in the future that will benefit patients with undesirable results from surgical misadventures where local tissues are not available for reconstruction. The authors conclude that despite many significant advances, the search for a suitable tissue engineering approach for use in routine clinical applications continues.

A vital part of successful correction of severe hypospadias is the straightening of the penis. Although not included in this research topic, a recent article in *Frontiers* provides a comprehensive review of this topic and is highly recommended for surgeons interested in this topic (1).

## Author Contributions

All authors listed have made a substantial, direct and intellectual contribution to the work, and approved it for publication.

### Conflict of Interest Statement

The authors declare that the research was conducted in the absence of any commercial or financial relationships that could be construed as a potential conflict of interest.
